# Patient safety in surgical environments: Cross-countries comparison of psychometric properties and results of the Norwegian version of the Hospital Survey on Patient Safety

**DOI:** 10.1186/1472-6963-10-279

**Published:** 2010-09-22

**Authors:** Arvid S Haugen, Eirik Søfteland, Geir E Eide, Monica W Nortvedt, Karina Aase, Stig Harthug

**Affiliations:** 1 Department of Anaesthesia and Intensive Care, Haukeland University Hospital, Jonas Lies vei 65, N-5021 Bergen, Norway; 2 Centre for Clinical Research, Haukeland University Hospital, Jonas Lies vei 65, N-5021 Bergen, Norway; 3 Department of Public Health and Primary Health Care, University of Bergen, Kalfarveien 31, N-5018 Bergen, Norway; 4 Centre for Evidence Based Practice, Bergen University College, P.O. Box 7030, N-5020 Bergen, Norway; 5 Faculty of Social Sciences, University of Stavanger, N-4036 Stavanger, Norway; 6 Department of Medicine, University of Bergen, P.O. Box 7804, N-5020 Bergen, Norway; 7 Department of Research and Development, Haukeland University Hospital, Jonas Lies vei 65, N-5021 Bergen, Norway

## Abstract

**Background:**

How hospital health care personnel perceive safety climate has been assessed in several countries by using the Hospital Survey on Patient Safety (HSOPS). Few studies have examined safety climate factors in surgical departments per se. This study examined the psychometric properties of a Norwegian translation of the HSOPS and also compared safety climate factors from a surgical setting to hospitals in the United States, the Netherlands and Norway.

**Methods:**

This survey included 575 surgical personnel in Haukeland University Hospital in Bergen, an 1100-bed tertiary hospital in western Norway: surgeons, operating theatre nurses, anaesthesiologists, nurse anaesthetists and ancillary personnel. Of these, 358 returned the HSOPS, resulting in a 62% response rate. We used factor analysis to examine the applicability of the HSOPS factor structure in operating theatre settings. We also performed psychometric analysis for internal consistency and construct validity. In addition, we compared the percent of average positive responds of the patient safety climate factors with results of the US HSOPS 2010 comparative data base report.

**Results:**

The professions differed in their perception of patient safety climate, with anaesthesia personnel having the highest mean scores. Factor analysis using the original 12-factor model of the HSOPS resulted in low reliability scores (r = 0.6) for two factors: "adequate staffing" and "organizational learning and continuous improvement". For the remaining factors, reliability was ≥ 0.7. Reliability scores improved to r = 0.8 by combining the factors "organizational learning and continuous improvement" and "feedback and communication about error" into one six-item factor, supporting an 11-factor model. The inter-item correlations were found satisfactory.

**Conclusions:**

The psychometric properties of the questionnaire need further investigations to be regarded as reliable in surgical environments. The operating theatre personnel perceived their hospital's patient safety climate far more negatively than the health care personnel in hospitals in the United States and with perceptions more comparable to those of health care personnel in hospitals in the Netherlands. In fact, the surgical personnel in our hospital may perceive that patient safety climate is less focused in our hospital, at least compared with the results from hospitals in the United States.

## Background

Patient safety climate in hospitals has recently gained greater attention. The Hospital Survey on Patient Safety (HSOPS) has been used widely to measure the safety culture in hospitals in the United States since it was introduced in 2004 [1,2]. The HSOPS is translated to 17 languages and used in 30 countries [3]. The psychometric properties of the HSOPS have been assessed in the US, the Netherlands, United Kingdom and Norway in large-scale studies on safety attitudes among health care providers in hospitals with varying results [1,4-6]. The results from UK and the Netherlands suggest deviations from the 12 factor structure of the questionnaire and the reliability tests vary between the factors; ranging from r = 0.49 to r = 0.83 [4,5]. The previous psychometric evaluation of the Norwegian version of HSOPS presented a 12 factor structure of which one had very low reliability "organizational learning - continuous improving" (r = 0.51) and also five other factors had lower reliability (r < 0.7) than recommended [6]. Further, other results indicate that the outcome variable "number of events reported" is probably not useful as an outcome measure [5,7]. Except for these limitations, the validity of the Norwegian HSOPS version was satisfactory regarding the confirmative factor analysis and construct validity [6,8]. The study was performed at one teaching hospital and concluded that further studies of the questionnaires psychometric properties is required, subsequently in Norwegian university hospitals to gain more knowledge of these properties [6].

The HSOPS can be regarded as measuring the patient safety climate giving a picture of the safety culture at a specific time point. The developers of the survey recommend not using the word "culture" as it tends to be confused with ethnicity or race [2]. Studies focusing on safety climate in the fields of surgery or anaesthesia have previously been performed using tools like the Safety Attitudes Questionnaire; however, for the HSOPS, the safety climate of operating theatre personnel has not been focused as one environment [1,2,4-11].

Operating theatres can be described as being units of high complexity and hazard with high potentials for patient harm and adverse events. Adverse events occur in 2.9-16.6% of admitted hospital patients, many of these (37-51%) probably being preventable [12-20]. More than half of all adverse event cases (51-62%) are associated with surgical services [19,21,22]. De Vries *et al*. categorize the types of adverse events as operation- or drug- related and majority of these events are located at the operating theatres [21]. Safety attitude instruments presents relationship to patient outcomes as correlations to fewer medical errors [23,24]. Promoting high reliability care in the surgical environment as the operating theatre needs a strong patient safety climate [25,26].

The objective of this study was to examine psychometric properties of the Norwegian HSOPS and compare our results to comparative database results from hospitals in the United States and to results from the Netherlands and Norway.

## Methods

### Design

This study was a cross-sectional survey examining perceptions of patient safety climate in operating theatre personnel using the validated Norwegian version of the HSOPS [1,5,6]. The Norwegian version of the HSOPS has previously been validated for paper distribution [7]. The questionnaire was translated into Norwegian before it was retranslated back to English, processed by two independent researchers. A pilot test was performed using health care personnel, to ensure that the concepts were correctly worded and conceptualized [8]. We performed the survey using a mixed distribution method, with a web and a paper version of the HSOPS. Before we distributed the survey, eight health care workers and research personnel pilot tested the readability and functionality of the web-based version. This pilot test resulted in splitting the first section of the HSOPS into two separate sections to improve readability. The Norwegian version has not previous been validated using a mixed distribution method.

### Sample

The sample consisted of operating theatre personnel at Haukeland University Hospital, Bergen, Norway: surgeons, anaesthesiologists, operating theatre nurses, nurse anaesthetists and ancillary personnel (unit assistants, clerks and cleaning assistants) present at work during a four week study period in October and November 2009. The hospital is one of the largest in Norway with 1,100 beds and about 10,000 employees, serving a population of 950,000 as a referral hospital and 500,000 as an emergency hospital. The annual number of surgical procedures exceeds 24,000. The following surgical departments were included in the survey: orthopaedic; thoracic; neuro-; ear, nose and throat; maxillofacial; plastic; endocrine; urinary; gastrointestinal; and obstetric surgery. Of the eligible personnel 575 individuals were invited to participate. Thirty-one enlisted personnel were absent during the study period due to vacation, illness, working other places, education or specialist training and were not included.

Selection of the clinical setting of this sample presents a large number of physicians; surgeons and anaesthesiologists, compared to specialist nurses contrasting other patient safety climate studies which included all health care personnel of the hospitals [5-7,10,11]. The operating theatre personnel are located at three separate locations: the largest of them are the central operating unit with 19 operating theatres, the women's clinic with 5 operating theatres in a separate building, and the day surgical unit with 2 operating theatres being physical connected to the central operating unit.

### Data collection

We distributed the web-based questionnaire to the operating theatre personnel through the hospital e-mail system. A paper version was sent to the personnel not responding to e-mail reminders. Physicians received two e-mail reminders before being sent the paper version. The operating theatre nurses, nurse anaesthetists and ancillary personnel received the paper version after one reminder. Some of the operating theatre personnel had logistical and technical difficulties in responding to the web-based questionnaire, such as being unable to gain access to the web version when using common log-on procedures and not having enough time in between daily routines. Identification numbers were assigned to or printed on each questionnaire to identify the working area or unit. We preserved the anonymity of data collection for the paper version by having respondents use closed envelopes addressed to the primary investigator (ASH) through the hospital's internal mail system. A consultant at the hospital research and development department administered the web-based questionnaire.

### Data screening

We examined data and checked for errors. Respondent who answered less than half the questionnaire items were excluded. Five respondents had chosen two options in one item, and we allocated these to the most positive or negative value of the categories [27]. The highest number of missing values was in the factor "frequency of events reported", which had missing values in 5.3% of the items. In the remaining factors, missing values were present in 0.3% to 3.1% of the cases. We did not exclude any items based on these few missing values and replaced them by the mean scores of the item.

### Questionnaire

Westat developed the HSOPS for the United States Agency for Healthcare Research and Quality as a safety culture assessment tool. Patient safety culture factors were selected based on a literature review of research pertaining to safety, error and accidents and an examination of previously existing safety culture assessment tools [27]. During the development of the HSOPS, hospital employees and administrators were interviewed to identify key issues related to patient safety and error reporting. The factors and items finally included in the HSOPS were selected to reveal information on relevant safety topics and to ensure satisfactory psychometric properties [6,24,26,27]. The HSOPS displays the perceptions of patient safety climate in 12 factors (Table 1). The patient safety climate factors contain three or four items each (a total of 42 items) and are all measured on a Likert scale, with a score from 1 to 5 on level of agreement: strongly disagree (1), disagree (2), neutral (3), agree (4) and strongly agree (5) [2]. The HSOPS also comprises two single-item outcome measures:

**Table 1:** Patient safety climate factors of the HSOPS used in the HSOPS study at Haukeland University Hospital, Bergen, Norway in October-November 2009

Patient safety climate factors of the HSOPS		Items
1.	Overall perception of safety	4

2.	Frequency of events reported	3

3.	Supervisor or manager expectations and actions promoting patient safety	4

4.	Organizational learning - continuous improvement	3

5.	Teamwork within units	4

6.	Communication openness	3

7.	Feedback and communication about error	3

8.	Non-punitive response to error	3

9.	Adequate staffing	4

10.	Hospital management support for patient safety	3

11.	Teamwork across hospital units	4

12.	Hospital handoffs and transitions	4

• the patient safety grade, scored from 1 to 5; failing (1), poor (2), acceptable (3), very good (4) or excellent (5); and

• the number of adverse events reported by the respondent during the last year, scored from 1 to 6; no events (1), 1-2 events (2), 3-5 events (3), 6-10 events (4), 11-20 events (5) and ≥ 21 events (6).

Sample characteristics are included such as profession, clinical experience, working hours during the week and working area or unit. Results are compared with data from 885 United States hospitals, the Hospital Survey on Patient Safety: 2010 User Comparative Database Report, as well as data from three hospitals in the Netherlands and one university hospital in Norway [2,5,6,8]. The results are presented as percent of average positive response (agree or strongly agree) in each item and factor, the highest percentage being the most positive. Sorra et al. [2] describe the method of calculation. In the previous psychometric evaluation study of the Norwegian version of the HSOPS, the twelve factors were classified as outcome variables (factor 1 and 2), measures at hospital unit level (factor 3-9) and at an overall hospital level (factor 10-12) [6].

### Statistical analysis

Descriptive statistics was used to display the frequencies of sample characteristics and patient safety climate factors. Negatively worded items were reversed to ensure that positive answers indicated a high score. For the 12 factors of the HSOPS questionnaire inferential statistics were used. To analyse differences in the means of explanatory variables according to profession and surgical departments' one-way analysis of variance was used. To investigate whether the HSOPS would fit with the data from a surgical environment sample in Norway we performed factor analysis using Varimax rotation [28]. Bartlett's test was used to examine if the inter-item correlations were sufficient. The chi-square distribution should correspond with the significance level of *P *= 0.05 [29]. The Kaiser-Meyer-Olkin (KMO) measure of sampling adequacy, with value range from 0 to 1, should exceed 0.5 to meet Kaiser's criterion [30]. The internal consistency of the factors was assessed by intra-class correlations and by Cronbach's alpha. For the factors to be consistent the alphas should exceed 0.7 [31]. We measured correlations by Pearson's correlation coefficient and internal consistency by Cronbach's alpha. We used SPSS (version 17.0) for Windows for data analysis [32].

### Ethics

The study was performed according to the ethical standards of the Helsinki Declaration [33]. The hospital research manager and the unit management leaders approved the study. The Committee for Medical Research Ethics of Western Norway reviewed the study and responded that approval was not necessary according to Norwegian law, since the study did not involve patients. The data privacy unit at Haukeland University Hospital consented to the project.

## Results

### Sample

The final sample included 575 operating theatre personnel. The overall response rate (*n *= 358) for the survey was 62% (358/575) and, for each profession: surgeons 56% (126/225), anaesthetists 62% (47/76), operating theatre nurses 61% (84/138), nurse anaesthetists 84% (62/74) and ancillary personnel 63% (39/62). Physicians represented 48% of the respondents, nurses 41% and ancillary personnel 11%. Ninety-four percent of the personnel had been in direct contact with patients. Among the operating theatre personnel, 54% worked more than 37 hours, 41% worked 20-37 hours and 4% worked less then 20 hours per week. Forty-two percent of the respondents were male and 58% female. The participants responded using the web version in 59% of the cases, and 41% used the paper version as their final entry. Table 2 lists the sample characteristics.

**Table 2:** Characteristics of 358 respondents to the HSOPS in Haukeland University Hospital, Bergen, Norway, October-November 2009

Characteristics (*n*)	Category	*n *	(%)
Professions (*n *= 358)	Senior physician^a^	96	(26.6)

	Physician^a ^> 2 years experience	52	(14.6)

	Physician^a ^< 2 years experience	18	(5.0)

	Operating theatre nurse	68	(19.1)

	Nurse anaesthetist	74	(20.8)

	Ancillary personnel^b^	26	(7.2)

	Administration, unit level	24	(6.7)

	Missing	1	

Years at this hospital (*n *= 352)	< 1 year	17	(4.7)

	1-5 years	84	(23.5)

	6-10 years	67	(18.7)

	11-15 years	67	(18.7)

	16-20 years	43	(12.0)

	≥ 21 years	74	(20.7)

	Missing	6	

Years in profession (*n *= 349)	< 1 year	10	(2.9)

	1-5 years	99	(28.4)

	6-10 years	105	(30.1)

	11-15 years	41	(11.7)

	16-20 years	27	(7.7)

	≥21 years	67	(19.2)

	Missing	9	

Hours per week (*n *= 355)	< 20 hours	16	(4.5)

	20-37 hours	145	(40.8)

	> 37 hours	194	(54.7)

	Missing	3	

Sex (*n *= 358)	Male	150	(41.9)

	Female	208	(58.1)

^a^Physician: surgeons and anaesthesiologists.

^b^Ancillary personnel: unit assistants, clerks and cleaning assistants.

### Background variables

Two of the 12 patient safety culture factors were considered outcome variables: "overall perception of safety" and "frequency of events reported". Seven of the remaining 10 factors were classified to be measured at the hospital unit level and the last three at the hospital level. The means of these factors were compared according to the background variables using one-way analysis of variance (ANOVA). Additional file 1: Table S1 presents the results for the various professions; the mean factor scores ranged from 2.80 to 3.55 between the five professional groups (ANOVA: *P *< 0.01). The mean factor scores of the two outcome variables differed between the professions (*P *< 0.01). In addition the mean factor scores differed within the variable "surgical departments" (ANOVA: *P *< 0.05) except for the factors "frequency of events reported" and "non-punitive response to error".

### Reliability and validity

The internal consistency of the patient safety climate factors was confirmed when measured using Cronbach's alpha, ranging from 0.64 to 0.85, except for the factor "adequate staffing", the internal consistency was 0.59. The correlations found supported discriminate and construct validity. The unit-level factors had mutual correlations ranging from 0.20 to 0.61 (*P *< 0.01). The hospital-level factors had correlations varying from 0.26 to 0.62 (*P *< 0.01). The correlation between the outcome variables "patient safety grade" and "overall perception of safety" was 0.59 (*P *< 0.01). Additional file 2: Table S2 lists all correlations.

### Factor analysis

Bartlett's test of the 42 patient safety climate items demonstrated a sufficient inter-item correlation: χ^2 ^= 6149; df = 946, *P *< 0.001. Further, the Kaiser-Meyer-Olkin measure of sampling adequacy was satisfactory, with a value of 0.91. Explorative factor analysis was performed using principal component analysis with Varimax rotation. Rotation converged after 11 iterations. Ten factors explained 60% of the total response variance. We compared the internal consistency measured by Cronbach's alpha with psychometric properties of the 2004 comparative database results from hospitals within the United States and the previous mentioned studies of the Netherlands and Norway (Table 3). For 10 of 12 factors, the Cronbach's alpha of our study was lower than those of the original factors from the United States data. Comparing the outcome variable "adequate staffing" resulted in unsatisfactory values on Cronbach's alpha ranging from 0.49 to 0.65. Combining the two factors "organizational learning and continuous improvement" with "feedback and communication about error" resulted in one factor with 6 items and an alpha value of 0.78.

**Table 3:** Cross-countries comparison of internal consistency of explorative factor analysis of the HSOPS

Explorative factor analysis						
**Patient safety culture factors of the HSOPS^a^**		**Items**	**United States **[35] **(*n *= 1437)** **Hospital environment** **Cronbach's α**	**Netherlands **[5] **(*n *= 3585)** **Hospital environment** **Cronbach's α**	**Norway** [8] **(*n *= 1919)** **Hospital environment** **Cronbach's α**	**Norway** **(*n *= 358)** **Operating environment** **Cronbach's α**

*Outcome variables*						

1.	Overall safety	4	0.74	0.62	0.76	0.78

2.	Frequency of events	3	0.84	0.79	0.82	0.82

*Unit-level factors*						

3.	Leader's expectations	4	0.75	0.70	0.79	0.85

4.	Continuous improvement	3	0.76	0.57	0.51	0.64

5.	Teamwork within units	4	0.83	0.66	0.77	0.75

6.	Open communication	3	0.72	0.72	0.68	0.67

7.	Error feedback	3	0.78	0.75	0.70	0.73

8.	Non-punitive	3	0.79	0.69	0.64	0.68

9.	Adequate staffing	4	0.63	0.49	0.65	0.59

*Hospital-level factors*						

10.	Management support	3	0.83	0.68	0.79	0.80

11.	Teamwork across units	4	0.80	0.68	0.65	0.73

12.	Handoffs and transitions	4	0.80	0.59	0.65	0.68

^a ^Complete labels: 1: overall perceptions of safety; 2: frequency of events reported; 3; supervisors' or managers' expectations and actions promoting patient safety; 4: organizational learning - continuous improvement; 5: teamwork within units; 6: communication openness; 7: feedback and communication about error; 8: non-punitive response to error; 9: adequate staffing; 10: hospital management support for patient safety; 11: teamwork across hospital units; 12: hospital handoffs and transitions.

### Comparative results

**Figure 1:**
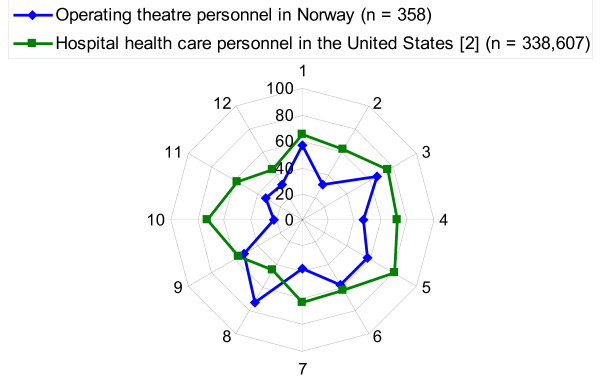
**Comparison of percent average positive responses of the HSOPS's patient safety climate factors between operating theatre personnel in Haukeland University Hospital in Norway October-November 2009 and hospital health care personnel in the United States (HSOPS 2010 user comparative database report)**. *Outcome variables*. 1. Overall perceptions of safety. 2. Frequency of events reported. *Unit-level factors*. 3. Supervisors' or managers' expectations and actions promoting patient safety. 4. Organizational learning - continuous improvement. 5. Teamwork within units. 6. Communication openness. 7. Feedback and communication about error. 8. Non-punitive response to error. 9. Adequate staffing. *Hospital-level factors*. 10. Hospital management support for patient safety. 11. Teamwork across hospital units. 12. Hospital handoffs and transitions

The percent of average positive responses (agree, strongly agree) varied between 22% and 72% across the twelve patient safety climate factors of the HSOPS. The total average percent of the average positive responses in all the patient safety climate factors was 47% in our sample. Table 4 compares these results to comparative database results from hospitals in the United States and results from the Netherlands and Norway. Figure 1 illustrates the variation between the percent average positive responses in the twelve patient safety climate factors of this study compared with the results from the United States.

**Table 4:** Cross-countries comparison of percent of average positive responses in patient safety climate factors of the HSOPS to responses from operating theatre personnel at Haukeland University Hospital in October-November 2009

		**United States **[2]	Netherlands^a^	**Norway **[8]	Norway
**Patient safety climate factors of the HSOPS^b^**		**Hospital environment** ***n *= 338,607**	**Hospital environment** ***n *= 3,779**	**Hospital** **environment** ***n *= 1,919**	**Operating environment** ***n *= 358**

		**%**	**%**	**%**	**%**

	*Outcome variables*				

1.	Overall safety	65	52	-	57

2.	Frequency of events	62	38	28	31

	*Unit-level factors*				

3.	Leaders' expectations	75	62	72	65

4.	Continuous improvement	72	47	50	46

5.	Teamwork within units	80	84	68	57

6.	Open communication	62	69	64	58

7.	Error feedback	63	49	40	37

8.	Non-punitive	44	67	72	72

9.	Adequate staffing	56	62	49	52

	*Hospital-level factors*				

10.	Management support	72	32	25	22

11.	Teamwork across units	58	28	31	32

12	Handoffs and transitions	44	40	39	31

Total average sum score		63	53	49	47

^a ^Source: Wagner C, Smits M. *Patient safety culture. Differences between professions and countries *http://internationalforum.bmj.com/2010-forum/presentation-slides/wednesday/A7%20Wagner,%20Smits.pdf

^b ^Complete labels: 1: overall perceptions of safety; 2: frequency of events reported; 3; supervisors' or managers' expectations and actions promoting patient safety; 4: organizational learning - continuous improvement; 5: teamwork within units; 6: communication openness; 7: feedback and communication about error; 8: non-punitive response to error; 9: adequate staffing; 10: hospital management support for patient safety; 11: teamwork across hospital units; 12: hospital handoffs and transitions.

## Discussion

### Variation in safety climate perception

The variation in the perception of patient safety climate factors between different surgical departments and between different professions was in accordance with previous HSOPS studies in Norway, which found significant differences (*P *< 0.001) both between clinical departments and professions [6,8]. Here we found significant variations (*P *< 0.05) in mean scores between different surgical departments regarding the patient safety climate at the outcome and unit factor levels. Between the different professions, the variation found was significant (*P *< 0.001) for the patient safety climate at the outcome and unit factor levels. The anaesthesiologists and nurse anaesthetists had higher mean scores than the surgeons and operating theatre nurses, both in outcome variables and unit-level factors. The ancillary personnel had the lowest mean scores here. The group with less education and being most distant to the patients, the ancillary personnel, reports most negative as to the patient safety climate. This might imply that perceptions of the safety climate may vary between operating theatre personnel groups. However, this needs further investigation to be validated.

The HSOPS is assumed to measure patient safety climate within hospitals and hospital units [2]. This is supported by our results and by findings in hospitals in the Netherlands, with clustering of responses within hospitals and hospital units rather than between individuals [34]. Our results may indicate variation in safety climate perceptions within surgical units and between operating theatre professions. The findings of our study may also reflect a perception of distance between the management at the unit and hospital levels regarding involvement in patient safety issues. Involvement and support from hospital management are strongly associated with the success of patient safety initiatives [35,36].

### Factor analysis

We used explorative factor analysis to investigate differences between our results and the comparative database results in the United States and results from the Netherlands and Norway [5,7,37]. The internal consistency of our data on operating theatre personnel was in-between those of the comparative studies, but the factor "organizational learning - continuous improvement" (α = 0.64) was more satisfactory in this study than in previous studies in the Netherlands and Norway [5-7]. Combining the factor "organizational learning - continuous improvement" with "feedback and communication about error" into an 11-factor structure produced internal consistency (Cronbach's α = 0.78) that was as satisfactory as for the study in the Netherlands [5]. In the psychometric evaluation of the HSOPS within a large acute National Health Service trust in the United Kingdom, more than half the factors failed to achieve satisfactory internal consistency (α < 0.7). Their factor analysis with split-half sample validation was converted into nine dimensions [4]. The results from the United Kingdom are interesting when considering this necessary psychometric evaluation of the questionnaire. They contrast somewhat with our findings in Norway. The correlations and patterns in our study seem more consistent in the construct validity of the patient safety climate factors compared with the previously mentioned studies [4-7]. The Norwegian version of the HSOPS cannot be regarded as externally validated until more Norwegian surgical environments and hospitals have been surveyed and the results compared and validated against patient outcomes.

### Comparative results

The health care personnel in United States hospitals generally seem to have a more positive perception of their hospitals' patient safety climate than operating theatre personnel in Norway. The largest difference in patient safety climate factors was found for the factor "hospital management support for patient safety", with a maximum 50 percentage-point difference in responses. These results, with the United States hospital personnel responding more positively, can be explained by cultural and organizational differences. Previous studies from Norway support our findings [6,8]. One explanation of the excessive variation could be that the owners of hospitals in Norway measure hospital managers not as much on patient safety as on financial results. Another major deviation from the United States 2010 User Comparative Database Report results in our study is for the factor "non-punitive response to error". The difference in positive responses is 28 percentage points, this time with the operating theatre personnel in Norway responding more positively. According to the Institute of Medicine of the United States National Academies, achieving a patient safety climate in which individuals are not blamed for errors (a non-punitive climate) may accomplish an important goal towards a safer health system [38]. Our results suggest that the surgical environment in our hospital seems to have a more non-punitive climate, although the "frequency of events reported" is 31 percentage points lower than in United States hospitals compared. The patient safety climate factor that correlated most strongly with this factor was "feedback and communication about error". We interpret that this may indicate that health care personnel experiencing feedback and communication about the errors reported would benefit the patient safety climate, giving incentives to the health care personnel to report events more frequently.

Our hospital has been using an electronic error reporting system for a relatively short time (3 years), and altering systems of error reporting may influence the frequency of reports. The difference in events reported between the hospitals in the United States and the HSOPS studies in Norway could also indicate a difference in cultural patterns. Our findings of low scores on "hospital management support in patient safety issues" may indicate that such tools as event reports and feedback on such reports should be used more extensively to motivate reports even further.

The perception of patient safety climate of our surgical environment and the hospitals in the studies previously mentioned seem to differ from those of the hospitals in the United States. Although there are minor differences in the factor structure, the variation in average positive responses of the twelve HSOPS factors, indicate differences in perceptions of the climate [4-6]. In fact, the surgical personnel in our hospital may perceive that this particular surgical environment has a lenient attitude towards patient safety climate. Low hospital management support results in low reporting of errors and a subsequent low frequency of feedback to the surgical units and personnel. This, together with few or no punitive measures, may create a low standard of patient safety as a final result.

### Limitations of the study

Several questionnaires are used worldwide to measure patient safety culture or climate, including the "Patient Safety Climate in Healthcare Organizations" [39], the "Culture of Safety Survey" [40], the "Safety Attitudes Questionnaire" [41] and the Hospital Survey of Patient Safety [1,2]. Evaluation of the psychometric properties of safety culture instruments have been performed in various ways [24,26]. Generally, these instruments measure abstract phenomena termed factors or dimensions from self reported perceptions of safety culture or safety attitudes. Such factors are by Byrne defined as indicators of the underlying construct they are presumed to represent. The use of sound psychometric instruments is then even more critical when the items measured are presumed to represent an underlying construct or factor [42]. When interpreting patient safety climate surveys one should have this limitation in mind.

This study is carried out in a single hospital, which limits the external validity of it even though the results are quite similar to the previous Norwegian studies [6,7]. The largest respondent group was the surgeons, who also had the lowest overall response rate (56%). Although the investigators persisted in informing the personnel about the survey, several respondents may have missed out on the information. We have not performed an analysis on nonresponders and cannot rule out the possibility of bias of variations in the mean scores between the professions. The average numbers of respondents in the studies compared varied from 37% to 56%; our overall response rate was 62%, however a response rate exceeding 70% would have been favourable [2,4,6,7].

## Conclusions

The psychometric properties of the Norwegian version of the HSOPS needs further investigation in surgical environments to be regarded as an appropriate instrument for assessing the patient safety climate among operating theatre personnel in large hospitals in Norway. The factor structures of the HSOPS questionnaire used in the United States, the Netherlands and Norway have minor differences. All originally defined items could be used, and internal consistency became more acceptable with the two factors "organizational learning - continuous improvement" and "feedback about and learning from error" combined into one six-item factor, supporting an 11-factor model. We found that professions and surgical departments differed in the perception of patient safety culture, but mainly the health care personnel in the United States and the surgical environments in Haukeland university hospital, differed regarding the patterns of patient safety climate. In fact, the operating theatre personnel in our hospital may perceive that patient safety climate is less focused in our hospital, at least compared with the results from hospitals in the United States.

## Competing interests

The authors declare that they have no competing interests.

## Authors' contributions

All authors contributed to the design and execution of the study. ASH, ES and SH conceived of and designed the study. ASH performed the data collection and drafted the manuscript. ASH and GEE performed the data analysis and all the authors contributed to the interpretation. ES, GEE, MWN, KA and SH contributed to and revised the manuscript critically for intellectual content. All authors read and approved the final draft.

## Pre-publication history

The pre-publication history for this paper can be accessed here:

http://www.biomedcentral.com/1472-6963/10/279/prepub

## Supplementary Material

Additional file 1 **Table S1: Patient safety climate factors according to profession in a large operating theatre environment at Haukeland University Hospital in October-November 2009: one-way analysis of variance of means**. Table S1 presents the results of one-way analysis of variance of means according to profession and patient safety climate factors. Click here for file

Additional file 2 **Table S2: Descriptive statistics^a^, intra-class correlations^b ^and correlations^c ^for outcome variables and sub dimensions of the HSOPS from the operating theatre personnel (*n *= 358) at Haukeland University Hospital in October-November 2009**. Table S2 presents the results of correlations between the patient safety climate factors. Click here for file
