# Supplementary material for: Patient safety in surgical environments: Cross-countries comparison of psychometric properties and results of the Norwegian version of the Hospital Survey on Patient Safety
Source: BMC Health Serv Res. 2010 Sep 22;10:279. doi: 10.1186/1472-6963-10-279 (PMC2955019; doi:10.1186/1472-6963-10-279)
Supplement: Additional file 1 — Table S1: Patient safety climate factors according to profession in a large operating theatre environment at Haukeland University Hospital in October-November 2009: one-way analysis of variance of means. Table S1 presents the results of one-way analysis of variance of means according to profession and patient safety climate factors. [file 1472-6963-10-279-S1.DOC]

**Additional file 1**

| **Table S1: Patient safety climate factors according to profession in a large operating theatre environment at**  **Haukeland University Hospital in October–November 2009: one-way analysis of variance of means** | | | | | | | | | | | | |
| --- | --- | --- | --- | --- | --- | --- | --- | --- | --- | --- | --- | --- |
|  |  | **Profession** | | | | | | | | | | |
| **Patient safety climate factors^a^** | **All**  ***n* = 358** | **Anaesthetists**  ***n* = 47** | | **Operating theatre nurses**  ***n* = 84** | | **Surgeons**  ***n* = 126** | | **Nurse anaesthetists**  ***n* = 62** | | **Ancillary personnel**  ***n* = 39** | | **Total**  ***n* = 358** |
|  | **Mean** | **Mean** | **95% CI** | **Mean** | **95% CI** | **Mean** | **95% CI** | **Mean** | **95% CI** | **Mean** | **95% CI** | ***P*** |
| *Outcome variables* |  |  |  |  |  |  |  |  |  |  |  |  |
| 1 Overall safety | 3.55 | 3.63 | 3.45–3.81 | 3.48 | 3.35–3.62 | 3.62 | 3.50–3.73 | 3.65 | 3.53–3.77 | 3.24 | 3.01–3.46 | 0.007 |
| 2 Frequency of events | 2.80 | 3.22 | 3.01–3.44 | 2.66 | 2.49–2.83 | 2.61 | 2.48–2.74 | 3.12 | 2.93–3.32 | 2.64 | 2.72–2.88 | <0.0001 |
| *Unit-level factors* |  |  |  |  |  |  |  |  |  |  |  |  |
| 3 Leader's expectations | 3.64 | 3.77 | 3.54–4.01 | 3.58 | 3.41–3.75 | 3.73 | 3.59–3.86 | 3.85 | 3.68–4.03 | 2.96 | 2.66–3.26 | <0.0001 |
| 4 Continuous improvement | 3.34 | 3.54 | 3.37–3.72 | 3.37 | 3.24–3.50 | 3.23 | 3.12–3.34 | 3.53 | 3.36–3.70 | 3.07 | 2.78–3.36 | 0.001 |
| 5 Teamwork within units | 3.59 | 3.80 | 3.64–3.96 | 3.44 | 3.32–3.55 | 3.66 | 3.54–3.78 | 3.73 | 3.58–3.88 | 3.21 | 3.05–3.37 | <0.0001 |
| 6 Open communication | 3.58 | 3.66 | 3.52–3.80 | 3.60 | 3.47–3.73 | 3.63 | 3.50–3.75 | 3.75 | 3.60–3.90 | 3.00 | 2.78–3.22 | <0.0001 |
| 7 Error feedbacks | 3.17 | 3.21 | 3.09–3.37 | 3.20 | 3.03–3.37 | 3.11 | 3.00–3.23 | 3.48 | 3.28–3.69 | 2.72 | 2.49–2.95 | <0.0001 |
| 8 Non-punitive | 3.74 | 4.05 | 3.88–4.23 | 3.75 | 3.62–3.88 | 3.77 | 3.63–3.89 | 4.00 | 3.87–4.12 | 3.00 | 2.73–3.28 | <0.0001 |
| 9 Adequate staffing | 3.38 | 3.55 | 2.77–3.22 | 3.31 | 3.17–3.45 | 3.45 | 3.32–3.63 | 3.49 | 3.34–3.63 | 2.98 | 2.78–3.18 | <0.0001 |
| *Hospital-level factors* |  |  |  |  |  |  |  |  |  |  |  |  |
| 10 Management support | 2.81 | 2.99 | 2.77–3.22 | 2.74 | 2.61–2.87 | 2.93 | 2.79–3.08 | 2.68 | 2.51–2.85 | 2.62 | 2.29–2.94 | 0.03 |
| 11 Teamwork across units | 3.12 | 3.08 | 2.93–3.23 | 3.09 | 2.98–3.20 | 3.22 | 3.11–3.32 | 3.06 | 2.95–3.16 | 3.08 | 2.92–3.24 | 0.20 |
| 12 Handoffs and transitions | 3.04 | 2.93 | 2.74–3.12 | 3.00 | 2.88–3.12 | 3.11 | 2.99–3.23 | 3.09 | 2.97–3.21 | 2.89 | 2.71–3.06 | 0.20 |

^a^ Complete labels: 1: overall perceptions of safety; 2: frequency of events reported; 3: supervisors’ or managers’ expectations and actions promoting patient safety; 4: organizational learning – continuous improvement; 5: teamwork within units; 6: communication openness; 7: feedback and communication about error; 8: non-punitive response to error; 9: adequate staffing; 10: hospital management support for patient safety; 11: teamwork across hospital units; 12: hospital handoffs and transitions.

CI: confidence interval.
