# Supplementary material for: Patient safety in surgical environments: Cross-countries comparison of psychometric properties and results of the Norwegian version of the Hospital Survey on Patient Safety
Source: BMC Health Serv Res. 2010 Sep 22;10:279. doi: 10.1186/1472-6963-10-279 (PMC2955019; doi:10.1186/1472-6963-10-279)
Supplement: Additional file 2 — Table S2: Descriptive statisticsa, intra-class correlationsb and correlationsc for outcome variables and sub dimensions of the HSOPS from the operating theatre personnel (n = 358) at Haukeland University Hospital in October-November 2009. Table S2 presents the results of correlations between the patient safety climate factors. [file 1472-6963-10-279-S2.DOC]

# Additional file 2

| **Table S2: Descriptive statistics^a^, intra-class correlations^b^ and correlations^c^ for outcome variables and sub dimensions of the** **HSOPS from the operating theatre personnel (*n* = 358) at Haukeland University Hospital in October–November 2009** | | | | | | | | | | | | | | | | | |
| --- | --- | --- | --- | --- | --- | --- | --- | --- | --- | --- | --- | --- | --- | --- | --- | --- | --- |
|  | **Items** | **Mean** | **SD** | **Cronbach’s α** | **PSG** | **AE** | **1** | **2** | **3** | **4** | **5** | **6** | **7** | **8** | **9** | **10** | **11** |
| *Outcome variables* |  |  |  |  |  |  |  |  |  |  |  |  |  |  |  |  |  |
| Patient safety grade | 1 | 3.56 | 0.58 | – |  |  |  |  |  |  |  |  |  |  |  |  |  |
| Adverse events | 1 | 1.84 | 1.04 | – | 0.09 |  |  |  |  |  |  |  |  |  |  |  |  |
| 1 Overall safety | 3 | 3.55 | 0.62 | 0.78 | 0.59^*^ | 0.08 |  |  |  |  |  |  |  |  |  |  |  |
| 2 Frequency of events | 3 | 2.80 | 0.78 | 0.82 | 0.28^**^ | 0.30^**^ | 0.28^*^ |  |  |  |  |  |  |  |  |  |  |
| *Unit-level factors*  3 Leaders’ expectations | 4 | 3.64 | 0.80 | 0.85 | 0.45^**^ | 0.16^**^ | 0.53^**^ | 0.36^**^ |  |  |  |  |  |  |  |  |  |
| 4 Continuous improvement | 3 | 3.34 | 0.66 | 0.64 | 0.39^**^ | 0.13^*^ | 0.45^**^ | 0.41^**^ | 0.48^**^ |  |  |  |  |  |  |  |  |
| 5 Teamwork within units | 4 | 3.59 | 0.61 | 0.75 | 0.43^**^ | 0.16^**^ | 0.53^**^ | 0.30^**^ | 0.56^**^ | 0.49^**^ |  |  |  |  |  |  |  |
| 6 Open communication | 3 | 3.58 | 0.65 | 0.67 | 0.36^**^ | 0.14^**^ | 0.51^**^ | 0.37^**^ | 0.57^**^ | 0.40^**^ | 0.49^**^ |  |  |  |  |  |  |
| 7 Error feedback | 3 | 3.17 | 0.73 | 0.72 | 0.36^**^ | 0.25^**^ | 0.42^**^ | 0.50^**^ | 0.56^**^ | 0.51^**^ | 0.43^**^ | 0.61^**^ |  |  |  |  |  |
| 8 Non-punitive | 3 | 3.74 | 0.69 | 0.67 | 0.28^**^ | 0.18^**^ | 0.56^**^ | 0.23^**^ | 0.41^**^ | 0.22^**^ | 0.45^**^ | 0.49^**^ | 0.28^**^ |  |  |  |  |
| 9 Adequate staffing | 4 | 3.38 | 0.65 | 0.59 | 0.45^**^ | 0.03 | 0.49^**^ | 0.20^**^ | 0.40^**^ | 0.26^**^ | 0.41^**^ | 0.32^**^ | 0.28^**^ | 0.45^**^ |  |  |  |
| *Hospital-level factors*  10 Management support | 3 | 2.81 | 0.74 | 0.80 | 0.44^**^ | -0.05 | 0.46^**^ | 0.22^**^ | 0.39^**^ | 0.36^**^ | 0.30^**^ | 0.31^**^ | 0.33^**^ | 0.23^**^ | 0.38^**^ |  |  |
| 11 Teamwork across units | 3 | 3.12 | 0.52 | 0.68 | 0.35^**^ | 0.01 | 0.37^**^ | 0.10 | 0.27^**^ | 0.28^**^ | 0.32^**^ | 0.27^**^ | 0.18^**^ | 0.16^**^ | 0.26^**^ | 0.53^**^ |  |
| 12 Handoffs and transitions | 4 | 3.04 | 0.60 | 0.73 | 0.37^**^ | 0.02 | 0.44^**^ | 0.19^**^ | 0.31^**^ | 0.26^**^ | 0.31^**^ | 0.28^**^ | 0.20^**^ | 0.31^**^ | 0.39^**^ | 0.45^**^ | 0.62^**^ |
| ^*^*P* < 0.05 (2-tailed); ^**^*P* < 0.01 (2-tailed).  ^a^Mean, standard deviation = SD. ^b^Intraclass correlation by Cronbach’s alpha. ^c^Pearson correlation.  PSG: patient safety grade; AE: adverse events reported on the past 12 months; 1: overall perceptions of safety; 2: frequency of events reported; 3: supervisors’ or managers’ expectations and actions promoting patient safety; 4: organizational learning – continuous improvement; 5: teamwork within units; 6: communication openness; 7: feedback and communication about error; 8: non-punitive response to error; 9: adequate staffing; 10: hospital management support for patient safety; 11: teamwork across hospital units; 12: hospital handoffs and transitions. | | | | | | | | | | | | | | | | | |
